# Nitric Oxide-Mediated S-Nitrosylation of TSC2 Drives mTOR dysregulation across Shank3 and Cntnap2 Models of Autism Spectrum Disorder

**DOI:** 10.1038/s41380-026-03514-6

**Published:** 2026-02-25

**Authors:** Shashank Kumar Ojha, Maryam Kartawy, Wajeha Hamoudi, Manish Kumar Tripathi, Adi Aran, Haitham Amal

**Affiliations:** 1 Institute for Drug Research, School of Pharmacy, Faculty of Medicine, The Hebrew University of Jerusalem, 9112002 Jerusalem, Israel; 2 Department of Pediatrics, Shaare Zedek Medical Center, 9103102 Jerusalem, Israel; 3 Faculty of Medicine, Hebrew University of Jerusalem, 9112002 Jerusalem, Israel; 4 The Rosamund Stone Zander and Hansjoerg Wyss Translational Neuroscience Center, Boston Children’s Hospital, Harvard Medical School, Harvard University, Boston, 02115 MA USA

**Keywords:** Biochemistry, Neuroscience

## Abstract

Autism spectrum disorder (ASD) is a complex neurodevelopmental disorder characterized by core behavioral symptoms. We previously showed that nitric oxide (NO) plays a key role in ASD. However, the precise molecular mechanism through which NO acts via its posttranslational modification, S-nitrosylation (SNO), in ASD remains largely unknown. Emerging evidence, including our previous studies, suggests that the mechanistic target of the rapamycin (mTOR) signaling pathway plays a critical role in ASD pathophysiology. Our SNO-proteome systems biology analysis showed the enrichment of the mTOR pathway. In this study, we deciphered a novel mechanism of the cross talk between NO and mTOR pathway using two well-established mouse models as well as clinical samples of children with ASD. To assess changes in the SNO-proteome, we used the SNOTRAP method, revealing increased S-nitrosylation of tuberous sclerosis complex 2 (TSC2) in *Shank3*^Δ*4–22*^ and Cntnap2^(-/-)^ mutant mice. We proved that this modification led to the loss of TSC2 protein via ubiquitination, resulting in dysregulated mTOR signaling in both excitatory and inhibitory neurons. Pharmacological inhibition of neuronal nitric oxide synthase (nNOS) successfully prevented TSC2 S-nitrosylation, mTOR overactivation, and altered protein translation in ASD models, highlighting NO’s role in modulating mTOR function. To further validate the role of TSC2 S-nitrosylation in ASD, we generated a cysteine-to-serine mutation (C203S) in TSC2 to prevent its S-nitrosylation. Intracranial injection of the mutant TSC2 (C203S) in *Shank3*^Δ*4–22*^ mice in the prefrontal cortex prevented ASD-like behaviors, confirming the pathogenic role of NO-mediated TSC2 modification. Critically, analysis of clinical samples from children with ASD, including those with SHANK3 mutations and idiopathic ASD, revealed reduced TSC2 levels and increased mTOR signaling activity, further validating our findings. Collectively, this study uncovers a novel molecular mechanism by which S-nitrosylation disrupts TSC2 function, leading to aberrant mTOR signaling and ASD-like phenotypes. By revealing a unique SNO-TSC2-mTOR axis, our work deciphers the novel nitric oxide-mediated mTOR activation and opens new avenues for targeted therapeutic strategies in ASD, including those carrying *SHANK3* mutations.

## Introduction

Autism spectrum disorder is a complex neurodevelopmental disorder, characterized by irregular social interaction, difficulty in communication, limited areas of interest, and repetitive behavior [1, 2]. The prevalence rate of ASD is increasing every year, i.e., 1/36 children from the latest epidemiological study in the USA [3]. To note, there is no pharmacological treatment for ASD. The etiology of ASD is complex, involving both genetic and environmental contributions, with heritability estimates ranging between 40–80% [4, 5]. Over the past two decades, several theoretical frameworks have been proposed to explain the pathogenesis of ASD. These include synaptic dysfunction, altered cortical connectivity, excitatory/inhibitory (E/I) imbalance, immune activation, and dysregulated mTOR signaling [1, 6].

Among the high-confidence ASD risk genes is *SHANK3*, which is a scaffolding protein located in the postsynaptic density complex playing a crucial role in the development and function of synapses [7]. *SHANK3* is highly associated with ASD, and deletions or mutations in the *SHANK3* gene have been found in individuals with Phelan-McDermid Syndrome (PMS), over 50% of whom are diagnosed with the disorder. [8, 9]. Another well-established gene is Cntnap2 (Contactin-associated protein-like 2) associated with ASD, which encodes a protein called contactin-associated protein-like 2 also called CASPR2 [10]. Cntnap2 is a member of the neurexin family protein present at the node of Ranvier and interacts with other proteins to help in the flow of ions across the cell membrane and proper conduction of nerve impulses along the axons [10, 11]. Mutation in these genes (*Shank3*^Δ*4–22*^/*Cntnap2*^(-/-)^) leads to impaired social interaction, intellectual disability, anxiety-like behavior, and reduced interest in novel objects [12–14].

Among the lesser explored, yet increasingly compelling biological pathways in ASD is NO signaling. NO is a redox-active gaseous molecule that regulates multiple aspects of neuronal physiology, including neurotransmission, synaptic plasticity, and immune responses [15]. Elevated NO levels have been reported in postmortem brains of ASD individuals, along with increased 3-nitrotyrosine accumulation, indicative of nitrosative stress [16]. Elevated NO levels in the brain have been linked to neuroinflammation, a common feature of ASD [6, 16–18]. Furthermore, our group and others have previously proved the link between NO and ASD as well showed the increased levels of NO in ASD mouse models, as well as in the blood samples of ASD individuals [16, 18–20]. We have also investigated the role of NO in physiological and pathological neurological states, which emphasized its importance in the brain [19–31]. However, despite these converging findings, it remains unclear whether aberrant NO metabolism is a causal contributor to ASD or merely an epiphenomenon accompanying broader neurodevelopmental disturbances. This distinction is critical, as many NO-related studies rely on genetically altered ASD models, which raises the possibility that observed redox changes may be downstream of synaptic gene dysfunction. As the field moves toward identifying convergent molecular mechanisms, a key gap remains: elucidating how NO signaling directly interfaces with established ASD-related pathways, such as mTOR, in a manner that is functionally and causally relevant to disease progression.

One major mechanism through which NO mediates its biological effects is S-nitrosylation [21, 32], a post-translational modification in which NO is covalently attached to the sulphur of a cysteine residue in a protein-forming S-nitrosothiol (SNO) [32–34]. SNO is involved in different physiological functions, such as cell signaling, neurotransmission, autophagy, and proteostasis [32, 34, 35]. In contrast, aberrant S-nitrosylation reactions by excessive reactive nitrogen species (RNS) can lead to different pathological conditions via protein misfolding, mitochondrial fragmentation, synaptic dysfunction, apoptosis, and autophagy inhibition [34]. In our previous study we showed that the SNO-proteome analysis of the cortex of the *Shank3* mutant mice revealed the mechanistic target of rapamycin (mTOR) pathway as the most significant pathway affected by SNO [36]. mTOR is a serine/threonine kinase, which plays an essential role in multiple physiological functions in the CNS by affecting the synaptic and neuronal functions [37–42]. Given the importance of the mTOR, it is not surprising that pathological hyper- or hypo-activation of this signaling pathway is potentially associated with different neurological disorders Tuberous sclerosis complex(TSC), Fragile X syndrome (FXS), ASD, epilepsy, Alzheimer’s Disease (AD) [36, 43–45]. Growing clinical data on neurodevelopmental disorders points to the critical role of the mTOR signaling pathway in ASD pathology [45–48]. Meanwhile, previous studies have also reported that mutation in TSC2 is involved in the hyperactivation of mTOR [49–51]. The loss of this protein in Purkinje cells may result in autistic-like behavior in mice [52–55]. TSC2 is an upstream master negative regulator of the mTOR activity [56, 57]. It forms a heterodimeric complex with TSC1 that suppresses mTORC1 activity by inhibiting the small GTPase Rheb, an essential activator of mTORC1 [58, 59]. The inhibitory effect of TSC2 on mTOR signaling plays a critical role in synaptic and neuronal processes, and overactivation of this signaling pathway contributes to the pathology of ASD [40, 60, 61].

In this study, we uncovered a redox-based mechanism in ASD pathophysiology: S-nitrosylation of TSC2 at cysteine 203, promotes its ubiquitination and proteasomal degradation, thereby resulting in mTOR overactivation. This mechanism was consistently validated in both *Shank3*^*Δ4–22*^ and *Cntnap2*^*−/−*^ mouse models and further supported by translational findings from clinical ASD plasma samples. Pharmacological inhibition of nNOS using 7-nitroindazole (7-NI), as well as overexpression of a S-nitrosylation–resistant mutant TSC2-C203S, rescued TSC2 stability, normalized mTOR activity, and improved ASD-like behaviors. Our study reveals for the first time a new mechanism of a cross talk between NO, TSC2, mTOR pathway, and ASD. This mechanism deciphers the molecular process behind the mTOR overactivation in ASD models.

## Materials and methodology

### Materials

Primary antibodies, anti-p-mTOR (#5536), anti-mTOR (#2983), anti-p-RPS6 (#4858), anti-RPS6 (#2317S), anti-β-actin (#3700), Anti-NeuN (#94403), anti-TSC2 (#4308), IgG Isotype control (3900S), CaMKII-alpha (50049S) and secondary antibodies, Anti-Rabbit Alexa fluor 594 (#8889), Anti-mouse Alexa Fluor 488 (#4408), HRP-conjugated anti-rabbit (7076S), HRP conjugated anti-mouse (7074S), ProLong gold Antifade with DAPI (#8961), and protease phosphatase inhibitor cocktail (#5872) were purchased from Cell Signaling Technology (Danvers, MA, USA). Anti-p70 S6 kinase (ab2571), anti-MAP2 (ab11268), anti-MAP2 (AB5392), Goat Anti-Chicken Alexa Fluor 647 (AB-ab150175) were purchased from Abcam, Cambridge, UK. Anti-Herc1 (sc-393950), anti-ubiquitin (sc-8017) was purchased from Santa Cruz Biotechnology, Dallas, TX, USA. Anti-puromycin (MABE343), anti-parvalbumin (P3088) were purchased from Merck, Sigma. 7-Nitro-1H-indazole (Cat. No.: L07970.06) from thermos-scientific, L-NIL (Cat. No.:CS-7870) from Chemscene, L-NAME hydrochloride (Cat. No.: ALX-105-003-G025) from Enzo, SNAP (ID: AG0063E3) from Angene, TG-003 (T5575) from Sigma. Other general chemicals were purchased from Sigma Aldrich (St. Louis, MO, USA) and Bio-Rad Laboratories (Haifa, Israel).

### Animals

Animal experiments were conducted according to the guidelines established by the Institutional Animal Care and Use Committee and the Association for Assessment and Accreditation of Laboratory Animal Care International (IACUC-MD-20-16049-3 and IACUC-MD-24-17501-4). We obtained *Shank3*^*Δ4–22*^ (Strain #: 03 2169), *Cntnap2*^*(-/-)*^ (Strain #: 01 7482), and C57BL/6 J (Strain #: 000664) mice from the Jackson Laboratory in Farmington, CT, USA. The *Shank3*^Δ4*–*22^ mouse strain is distinguished by the deletion of exons 4-22 in the gene responsible for SH3 and multiple ankyrin repeat domains 3 (*Shank3*). *Shank3*^Δ4*–*22^ homo littermates were used in the experiments. We used WT littermates as controls for the Shank3 experiments. In the case of *Cntnap*2^(-/-)^ mice, C57BL/6 J mice served as the control group. For biochemistry and behavioral assays, we used male *Shank3*^*Δ4–22*^ and their control littermates, as well as *Cntnap2*^*(-/-)*^ mice and wild-type (WT) C57BL/6 J mice. The animals were housed at a room temperature of 23 °C, following a 12 h light/dark cycle, and provided with standard mouse chow and water ad libitum.

### Animal treatments

*Shank3*^Δ*4–22*^ KO (KO1) and *Cntnap2*^*(-/-)*^ KO (KO2) were treated with 7-NI at 80 mg/kg/day or L-NAME (50 mg/kg/day) or L-NIL (5 mg/kg/day) via intraperitoneal (IP) for 14 days. For the dose-dependent study, 7-NI was administered IP at doses of 10, 20, and 40 mg/kg/day to *Cntnap2*^*(-/-)*^ knockout mice for 14 days, followed by behavioral testing (see Supplementary fig. S11). Only male mice are used in the experiments. *Shank3*^Δ*4–22*^ KO (KO1) and *Cntnap2*^*(-/-)*^ KO (KO2) and their respective control littermates WT mice (WT1 and WT2) were also treated with the vehicle. WT2 (C57BL/6 J also indicated as WT mice) mice were treated with S-nitroso-N-acetylpenicillamine (SNAP, NO donor) at 20 mg/kg/day via IP for 14 days, and as a control WT2 mice were treated with vehicles. WT2 mice were also treated with TG003 at 10 mg/kg/day via IP for 14 days and as a control WT2 mice were treated with vehicle.

### Immuno-precipitation

Tissue/cells were lysed in the lysis buffer and centrifuged at 17,000 g for 30 min at 4 C to remove insoluble matter. The experiment was performed with the Immunoprecipitation Kit-Dynabeads Protein G according to the protocol(10007D). Immunoprecipitation was done for the TSC2, and then immunoblotting was done for the ubiquitin and Herc1. IgG isotype control is used as negative control.

### Western blotting

Snap-frozen tissue was homogenized in fresh RIPA buffer as previously described (30 mm HEPES, pH 7.4, 150 mm NaCl, 1% Nonidet P-40, 0.5% sodium deoxycholate, 0.1% sodium dodecyl sulfate, 5 mm EDTA, 1 mm Na_3_V0_4_, 50mmNaF, 1mmPMSF, 1% protease, and phosphatase inhibitors cocktail, pH 7.7) at 4 degrees with bullet blender for 3 times and 15 s each. The homogenate was centrifuged at 17000 g for 40 min at 4 degrees. The supernatant was collected, and the protein concentration was estimated to be using the Bicinchoninic Acid (BCA) Protein Assay (Sigma Aldrich). Protein samples were mixed with Laemmli sample buffer, resolved on SDS-PAGE gels, and transferred onto PVDF membranes. Membranes were blocked with 5% BSA or 5% skim milk in TBST for 2 h at room temperature and incubated with primary antibodies overnight at 4 °C. After washing, membranes were incubated with HRP-conjugated secondary antibodies for 1 h at room temperature. Protein bands were visualized using Clarity Max ECL substrate and imaged with a ChemiDoc system (Bio-Rad).

### Cell culture

Human neuroblastoma (SH-SY5Y) cells were obtained from the American Type Culture Collection (Manassas, VA, USA) and maintained in a 1:1 mixture of Ham’s F-12 and Dulbecco’s modified Eagle’s medium (DMEM) supplemented with 10% fetal bovine serum, 2mm l-glutamine, and 1% penicillin-streptomycin in a humidified atmosphere at 37 °C and 5% CO2. *SHANK3* KO SH-SY5Y cells are generated as described previously [62]. SH-SY5Y cells are differentiated with retinoic acid as described previously [63]. SH-SY5Y cells were treated with puromycin (5 μg/ml) for 30 min.

### Cell viability assay

The dose of 7-NI and SNAP was determined using a colorimetric 3-(4,5- dimethylthiazol-2-yl)-2,5-diphenyl-tetrazolium (MTT) assay (Sigma-Aldrich). 1 ×10^4^ SH-SY5Y cells were seeded in 96 well plates for sextuplicate for each dose. Treatment was given for 24 h, and viability was assessed.

### Primary cortical culture

Primary cortical neuronal cultures were derived from embryonic day 16.5-17.5 fetal mouse brains. The cortex isolated from embryonic mouse brains was placed in HBSS (Gibco) and dissociated mechanically with a 19 G needle/syringe 2 times. Then, dissociated tissue was treated with trypsin-EDTA solution 0.25% (Sigma-Aldrich-T4049) at 37˚C for 5 mins. Trypsin activities were neutralized with FBS, filtered with 70μm nylon membrane, and then centrifuged at 400 x g for 10 min. The cells were seeded on poly‑D‑lysine (P1024, Sigma‑Aldrich) coated glass or plastic‑bottom 22‑mm coverslip placed in 6 well plates (cell density was ~1,50,000‑2,00,000) for microscopic observation. Cells were cultured in neurobasal medium [neurobasal medium (Gibco), 2% B27 supplement, 2 mM L‑glutamine, 1% penicillin‑streptomycin (P/S), and incubated at 37˚C in a humidified incubator with 95% air and 5% CO2. On the second day of neuronal culture, half the media volume was replaced with the same volume of fresh neurobasal media with 2 μm cytosine-*β*-d-arbinofuranoside (C1768, Sigma-Aldrich) to avoid glial cell proliferation. The cells were grown for 14 days. Transfection was done on day 7 with the help of lipofectamine 2000 and treatment of SNAP was done 24 h before the fixation of cells.

### SNOTRAP

The tissue was homogenized mechanically in the lysis buffer (pH = 7.7) prepared with 100 mM HEPES, 1 mM EDTA, 0.1 mM neoporcupine, 20 mM Iodoacetamide, 1% Triton-X100, 1% SDS and 1% protease cocktail as described previously [33]. The samples were centrifuged at 17,000 g for 45 min at 4° C. The supernatant was collected, and protein concentrations were estimated using the BCA (Bicinchoninic Acid) protein assay (Sigma Aldrich). We proceeded further with 12 mg of protein in the experiment. We washed the samples with 50 mM HEPES in 10 K MWCO. We washed three times at 5000 g for 30 min at 10° C. The samples were labeled with 1.5 mM SNOTRAP dissolved in acetonitrile and HEPES (50:50) for 2 h in the dark. SNOTRAP labeled samples were washed 3 times with 50 mM HEPES at 10^0^C for 30 min in 10 K MWCO tubes. The SNO proteins were pulled out with the help of streptavidin beads. The beads were then washed with 5 different buffers 1) 100 mM ABC + 150 mM NaCl +1 mM EDTA + 0.05% SDS + 0.1% tritox-100 (pH 7.4), 2) 100 mM ABC + 150 mM NaCl+1 mM EDTA + 0.1% SDS. 3) 100 mM ABC + 150 mM NaCl +0.05% SDS. 4) 100 mM ABC, 5) 50 mM Hepes pH 7.7) two times each. The proteins were eluted from the beads with 10 mM TCEP prepared in 50 mM ABC at 37^0^C for 30 mins [33].

### Confocal microscopy of brain sections

Coronal sections of the brain in the cortical region (20um thick) were done for the staining process. The section was washed with fresh PBS and then blocked with blocking buffer (PBS containing 1% BSA, 1% goat serum, and 0. 1% triton-X100) for 1 h. Blocking is followed by overnight incubation with specific antibodies NeuN (1: 500), p-mTOR (1: 200), p70S6 Kinase (1:200) and TSC2 (1:200). Then, the sections were washed with PBS for 3 times 10 min each, followed by incubation with anti-rabbit Alexa fluor 594 (1:1000) and anti-mouse Alexa Fluor 488 (1:1000) and anti-chicken Alexa fluor 647 (1:1000) secondary antibodies for 1 h in the dark. After incubation with secondary antibodies, sections were washed with PBS 4 times for 10 min each time. Sections were mounted with anti-fade DAPI. Confocal images were captured using 40×/60×/100× with a Nikon confocal microscope and a sequential acquisition setting of 2024 × 1024. Each image was a z-series projection of 8-10 images. The depth intervals of each image were determined by instruments automatically. The mean fluorescence intensity was calculated using Image J software, as described previously [20].

### Confocal microscopy of cultured cells and neurons

Primary cells/ cell lines cultured on 22 mm/19 mm coverslips were washed three times with PBS and fixed with 4% paraformaldehyde for 15 mins at room temperature. The fixed cells were washed 3 times for 10 min each with PBS and incubated in the chilled methanol for 5 min at room temperature. After washing the cells three times with PBS, they were blocked with blocking buffer (PBS containing 1% BSA, 1% goat serum, and 0.1% triton-X100) for 1 h at room temperature and then incubated with the respective primary antibodies prepared in the blocking buffer overnight at 4 °C. Next day the cells were washed 3 times with PBS for 10 min each and then the cells were incubated with anti-rabbit Alexa fluor 594 (1:1000) and anti-mouse Alexa Fluor 488 (1:1000) secondary antibodies for 1 h, washed with PBS 4 times, and mounted with the anti-fade DAPI mounting media. Finally, the cells were observed under 40×/60×/100× using a Nikon confocal microscope.

### Site-directed mutagenesis

Lenti‑TSC2‑C203S was constructed by NEB, Inc. Briefly, TSC2‑C203S site‑directed mutagenesis was performed using NEB’s Q5^®^ Site-Directed Mutagenesis Kit (NEB #E0554) according to the manufacturer’s instructions. Briefly, the primer was made on NEB, and inverse PCR was performed to mutagenesis and amplify the plasmid. Mutagenesis was confirmed by sequencing.

### Stereotaxic viral Injection

Brain surgeries were conducted on 4–5-week-old mice for AAV9 injection. We used AAV9 vectors (AAV9-CBA-TSC2-T2A-EGFP) (Vector ID: VB240119-1103ddf, VB240115-1306jgh), expressing mutant TSC2 (C203S) under the control of the CBA promoter. The viral vectors were packaged and purified by VectorBuilder with a final titer of 7.57×10¹³ GC/mL. As a control, an empty AAV9 vector expressing only EGFP (AAV9-CMV-EGFP) and the titer, 1.46 ×10¹³ GC/mL was used. Injections were performed bilaterally into the prefrontal cortex as described. The mice were anesthetized by isoflurane in Somnoflo isoflurane chamber. After shaving the hair and washing the scalp with iodine tincture and ethanol, the mice were placed into a stereotaxic setup. Artificial eye ointment was applied to cover the eyes for protection purposes. A midline scalp incision of the mouse brain was made, and bregma was decided. A drill hole on the skull was created for virus injection into the prefrontal cortex (AP + 1.7 mm, ML: ±0.5–0.6 mm, DV: −1.6–−1.8 mm) on both sides of the midline. Mice were provided with wet food for the next 3 days. The virus was allowed to be expressed for the next 14 days, and then a behavioral and biochemical experiment was performed.

### Human participants and blood plasma isolation

The experimental procedures were conducted with approval from the Share Zedek Medical Center Institutional Review Board (Jerusalem, Israel; IRB# 0501-20-SZMC, original approval granted on 02 December 2020). Blood samples were provided by Dr Adi Aran. Blood samples were obtained from 8 (5 unknown + 3 *Shank3* loss of function (LOF)) children diagnosed with autism spectrum disorder (ASD) and 5 age and gender-matched neurotypical children without any neuropsychiatric conditions who attended regular education classes. Participant demographics and symptom severity information are detailed in Table S1 and S2, Supporting Information. Recruitment occurred through outpatient clinics and community advertisements in Jerusalem, Israel. The study adhered to approved protocols, relevant guidelines, and regulations, with informed consent obtained from all participants. Venous whole blood was collected between 10:00 AM and 2:00 PM from non-fasting participants into EDTA-coated tubes to prevent coagulation. Whole blood samples were centrifuged for 10 min at 3000 x g within 15 min from the blood draw. The plasma aliquots were frozen immediately at −80 °C until use.

### nanoLC-MS/MS Analysis

The MS analysis was conducted utilizing a Q Exactive Plus mass spectrometer (Thermo Fisher Scientific, Waltham, MA, USA) coupled online with a nanoflow UHPLC instrument, Ultimate 3000 Dionex (Thermo Fisher Scientific, Waltham, MA, USA), as detailed in our previous study. Data acquisition was performed using Xcalibur software (Thermo Scientific).

### Mass spectrometry data analysis

The mass spectrometry data were processed utilizing the Max Quant computational platform, specifically version 2.0.3.0, as outlined in our previous study. Max Quant was performed to label-free quantification (LFQ) algorithm to conduct relative protein quantification.

### Behavioral assay

All behavioral tests were assayed on 6- to 8-week-old male mice. The behavioral experiments were recorded, and data were automatically scored using Ethovision XT 16 (Noldus Information Technology BV), a video tracking software employing AI-assisted tracking. This software monitors mice movements across two dimensions (x, y). It records data from three body points: tail, center, and nose-analysis for the interaction duration tests of the mice as described previously [20]. The arena was cleaned with 5% virusol/ 70% ethanol between trials.

#### Open field test

Mice were placed in an open arena (60 ×60 cm) and allowed to explore freely for 5 min, and their position was continually monitored. On the first day (habituation), mice were individually placed in the arena’s center for free movement. The next day, the same procedure was repeated. The Ethovision XT system measured the total distance traveled and the velocity of the mice during the experiment. Post-testing, the arena was sanitized with a 70% alcohol/virusol solution.

#### Object recognition test

The test is performed to assess object and novel object recognition in mice. Experiments were performed in 2 days, on the first day (familiarization session for 5 min), the mice were left to explore two identical objects that could be found at constant locations, 15 cm from the sidewalls, in the already familiar white plastic arena. On the second day (test session), the mice were introduced to the arena in which one of the familiar objects was replaced by a novel object. Exploration by the mice was defined as directing their nose to the object at distance of ≤1 cm and/or making contact with the object using their nose. The duration of time spent by each mouse exploring the objects was recorded for 5 min. Both the arena and the objects were cleaned with a 70% alcohol solution after each session.

#### Elevated plus maze test

This test examines anxiety-like behavior in rodents. The platform consists of four arms (30 ×5 cm): two open and two closed, placed 45 cm above the floor. During the test, mice were placed in the center of the platform, facing one of the open arms, and letting it move freely. The test was performed for 10 mins in TG003 treated mice and for 5 min in stereotaxic experiments for each mouse. The time spent in the closed and open arms was recorded.

#### Three-chambered social test

A three-chamber sociability test was used to evaluate sociability and novelty-seeking behaviour. This experiment was performed as described previously [20].

#### Statistics and bioinformatics

The mean and Standard Error of the mean (SEM) were calculated for the western blots, immunofluorescence, and behavioral experiments. A one-way ANOVA test and the post-Tukey’s multiple comparison tests were used for the behavioral, immunofluorescence and western blot tests. The figure legends show the statistical details and methods used in each experiment. Bar graphs and statistical analysis were performed using Prism 9.3 (GraphPad Software, San Diego, CA). The data are presented as the mean + SEM. *p < 0.05, **p < 0.01, ***p < 0.001, and ns = not significant.

## Results

### Nitric oxide mediates overactivation of mTOR signaling in ASD mouse models

**Fig. 1:**
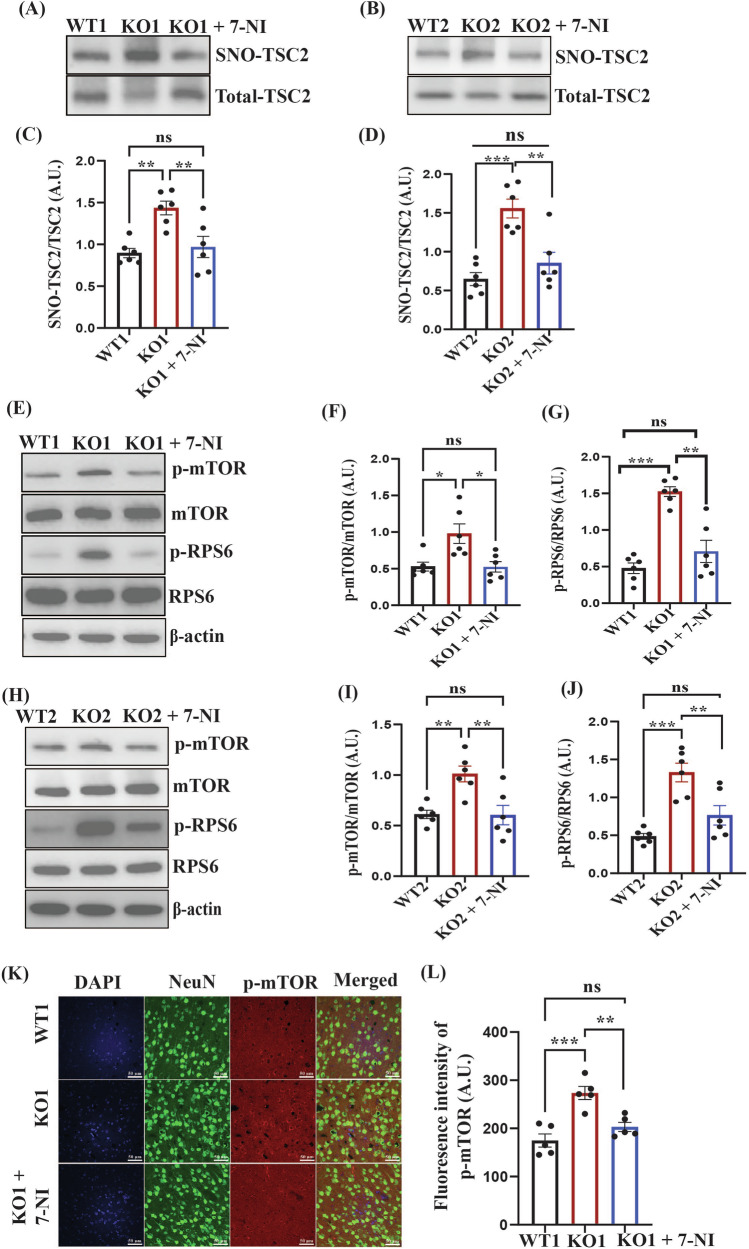
nNOS inhibition prevented aberrant S-nitrosylation of TSC2 and overactivation of mTOR signaling pathways. **A** Representative WB of SNO-TSC2 and total TSC2, in WT1, *Shank3*^Δ*4–22*^ KO (KO1), and *Shank3*^Δ*4–22*^ KO + 7-NI. **B** Representative WB of SNO-TSC2 and total TSC2, in WT2, *Cntnap2*^*(-/-)*^ KO (KO2) and *Cntnap2*^*(-/-)*^ KO + 7-NI. **C** Statistical analysis of the relative abundance of SNO-TSC2/TSC2 (n = 6) in WT1, *Shank3*^*Δ4–22*^ KO, and *Shank3*^Δ*4–22*^ KO + 7-NI. **D** Statistical analysis of relative abundance of SNO-TSC2/total TSC2 (n = 6) in WT2, *Cntnap2*^*(-/-)*^ KO and *Cntnap2*^*(-/-)*^ KO + 7-NI. **E** Representative WB images of p-mTOR, mTOR, p-RPS6 and RPS6 in WT1, *Shank3*^Δ*4–22*^ KO and *Shank3*^Δ*4–22*^ KO + 7-NI. β-actin was used as a loading control. **F-G** Statistical analysis of relative abundance of p-mTOR/mTOR (n = 6), p-RPS6/RPS6 (n = 6) in WT1, *Shank3*^Δ*4–22*^ KO and *Shank3*^Δ*4–22*^ + 7-NI. **H** Representative WB images of p-mTOR, mTOR, p-RPS6 and RPS6 in WT2, *Cntnap2*^*(-/-)*^ KO and *Cntnap2*^*(-/-)*^ KO + 7-NI. β-actin was used as a loading control. **I-J** Statistical analysis of relative abundance of p-mTOR/mTOR (n = 6), p-RPS6/RPS6 (n = 6) in WT2, *Cntnap2*^*(-/-)*^ KO and *Cntnap2*^*(-/-)*^ KO + 7-NI. **K** Representative confocal images of DAPI (blue), NeuN (Green), p-mTOR(red) in WT1, *Shank3*^Δ*4–22*^ KO and *Shank3*^Δ*4–22*^ + 7-NI cortex. The image was captured at 60x magnification. Scale bar = 50μm. **L** Statistical analysis of fluorescence intensity of p-mTOR (n = 5). Data are presented as mean ± SEM. For all the experiments statistical significance was determined by a one-way ANOVA test with Tukey’s multiple comparison’s test in all groups. *p < 0.05, **p < 0.01, ***p < 0.001, ns not significant.

Initially, we aimed to investigate whether NO signaling and S-nitrosylation (SNO) regulate TSC2 stability. To assess this, we measured TSC2 S-nitrosylation levels in wild-type (WT) and knockout (KO) mice using SNOTRAP labeling followed by immunoblotting. Our findings revealed a significant increase in TSC2 S-nitrosylation in *Shank3*^Δ*4–22*^ and *Cntnap2*^*(-/-)*^ mutant mice compared to their respective WT controls (Fig. 1A-D). Treatment of *Shank3*^Δ*4–22*^ and *Cntnap2*^(-/-)^ mice with 7-NI, a nNOS inhibitor, effectively prevented TSC2 S-nitrosylation in both mutants (Fig. 1A-D). In addition, we observed a decrease in total TSC2 levels in *Shank3*^Δ*4–22*^ and *Cntnap2*^(-/-)^ mutant mice compared to respective WT controls. However, administration of 7-NI significantly prevented increased S-nitrosylation of TSC2 and rescued total level of TSC2 in both models (Fig. 1A-D). To confirm NO dependence and exclude off-target effects, we pharmacologically inhibited distinct NOS isoforms in Shank3^Δ4*–*22^ KO mice (Supplementary fig. S6). nNOS blockade with 7-NI and non-selective NOS inhibition with L-NAME significantly restored cortical TSC2 toward WT levels, whereas the iNOS-selective inhibitor L-NIL produced no significant change relative to KO + vehicle. These data implicate nNOS-derived NO in the TSC2 reduction observed in the KO mice.

To further explore the downstream effects of NO inhibition, we analyzed mTOR signaling activation by quantifying phosphorylated mTOR (p-mTOR), a key indicator of pathway activation. Our results showed increased levels of p-mTOR and phosphorylated ribosomal protein S6 (p-RPS6) in the cortex of *Shank3*^Δ*4–22*^ and *Cntnap2*^(-/-)^ mutant mice relative to WT controls (Fig. 1E-J). Notably, treatment with 7-NI effectively suppressed mTOR overactivation in both ASD models, as evidenced by decreased levels of p-mTOR and p-RPS6 compared to vehicle-treated mutants (Fig. 1E-J). We have also found significant increased level of p-mTOR and p-RPS6 in the striatum of *Shank3*^Δ*4–22*^ and *Cntnap2*^(-/-)^ mice as compared to respective WT control while 7-NI treatment significantly reduced the level of p-mTOR and p-RPS6 in *Shank3*^Δ*4–22*^ and *Cntnap2*^(-/-)^ as compared to the vehicle treated group (Figures S1 and S2). To assess possible systemic or off-target effects of 7-NI, we treated WT mice daily for 14 days. No significant differences were observed in TSC2 or p-mTOR levels compared with vehicle-treated controls (Supplementary fig. S3). Body weights remained stable throughout the treatment period (Supplementary fig. S4), indicating that 7-NI did not affect basal mTOR signaling or general physiology. Additionally, fluorescence intensity analysis of p-mTOR in the cortical region of *Shank3*^Δ*4–22*^ mice demonstrated significantly increased activation compared to WT. However, 7-NI treatment in *Shank3*^Δ*4–22*^ mice led to a significant reduction in p-mTOR fluorescence intensity compared to vehicle-treated mutants (Fig. 1K, L). These findings indicate that NO plays a crucial role in modulating mTOR signaling.

### NO-mediated S-nitrosylation of TSC2

**Fig. 2:**
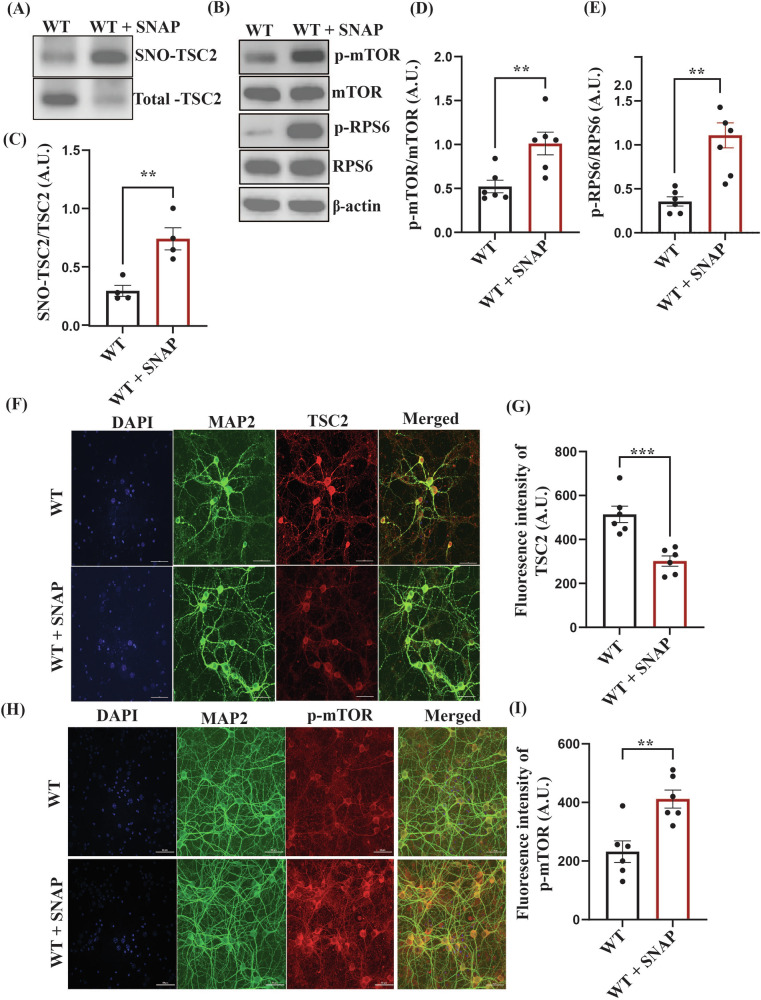
NO mediated SNO-TSC2. **A** Representative WB of SNO-TSC2 and total TSC2, in WT and WT + SNAP. **B** Representative WB images of p-mTOR, mTOR, p-RPS6 and RPS6 in WT and WT + SNAP. β-actin was used as a loading control. **C** Statistical analysis of relative abundance of SNO-TSC2/TSC2 (n = 4) in WT and WT + SNAP. **D-E** Statistical analysis of the relative abundance of p-mTOR/mTOR (n = 6), p-RPS6/RPS6 (n = 6) in WT and WT + SNAP. **F** Representative confocal images of DAPI (blue), MAP2 (Green), and TSC2(red) in WT, WT + SNAP cortical neurons. The image was captured at 60x magnification, scale bar = 50μm. **G** Statistical analysis of fluorescence intensity of TSC2 (n = 6). **H** Representative confocal images of DAPI (blue), MAP2 (Green), and p-mTOR(red) in WT, WT + SNAP cortical neurons. The image was captured at 60x magnification. Scale bar = 50μm (**I**) Statistical analysis of fluorescence intensity of p-mTOR (n = 6). Data are presented as mean ± SEM. An unpaired two-tailed Student’s T-test was performed. **p < 0.01, ***p < 0.001.

To further confirm the S-nitrosylation of TSC2 is NO-mediated, we treated WT mice with SNAP (NO donor) [64]. Using SNOTRAP [19], we have found that WT mice treated with SNAP led to the increased S-nitrosylation of TSC2 compared to WT mice treated with vehicle (Fig. 2A, C). We have also found a decreased level of total TSC2 in SNAP-treated WT mice compared to WT treated with vehicle (Fig. 2A, C). A decreased level of TSC2 fluorescence intensity in WT cortical neurons treated with SNAP as compared to the WT cortical neurons was found as well in the statistical analysis (Fig. 2F, G). To test if the activation of mTOR pathways is NO-mediated, we measured the level of p-mTOR in WT mice treated with SNAP and found an increased level of p-mTOR and p-RPS6 as compared to WT mice treated with vehicle (Fig. 2B, D, E). We have also found an increased level of p-mTOR intensity in WT cortical neurons treated with the SNAP compared to the WT cortical neurons treated with vehicle (Fig. 2H I). These results confirm that NO leads to SNO-TSC2 and to overactivation of the mTOR signaling pathway.

### nNOS inhibition rescues neuronal TSC2 levels and prevents its proteasomal degradation

**Fig. 3:**
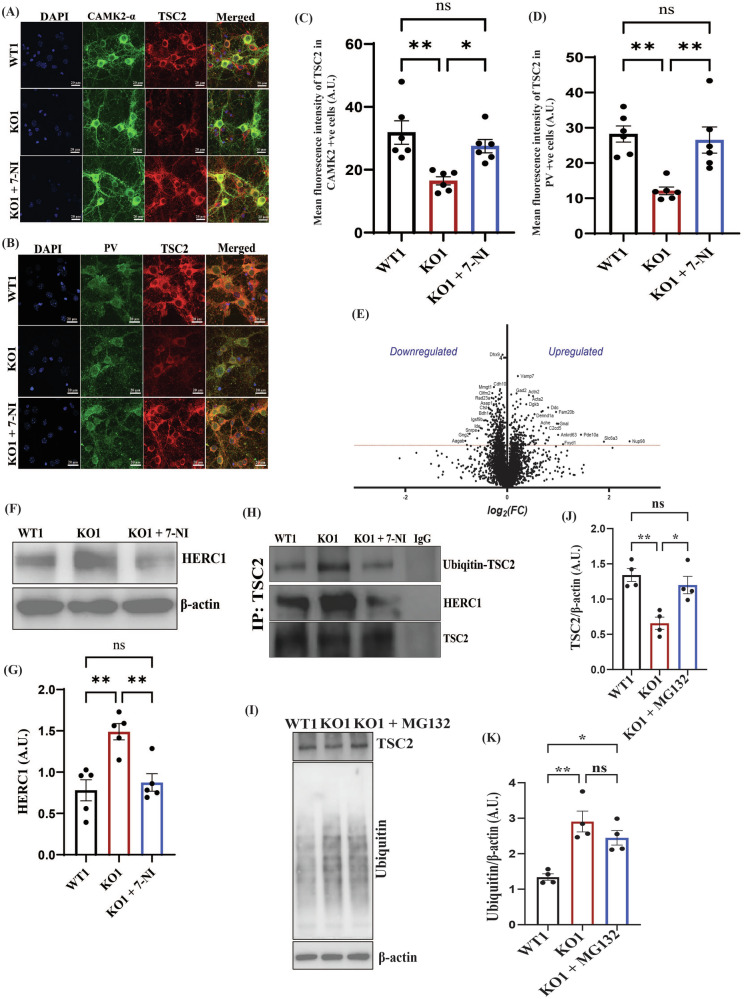
nNOS inhibition rescues neuronal TSC2 levels and prevents its proteasomal degradation. (**A**) Representative confocal images of DAPI (blue), Camk2-α (Green), TSC2 (Red) in WT1, *Shank3*^Δ*4–22*^ KO and *Shank3*^Δ*4–22*^ KO + 7-NI primary cortical neurons. **B** Representative confocal images of DAPI (blue), PV (Green), TSC2 (Red) in WT1, *Shank3*^Δ*4–22*^ KO and *Shank3*^Δ*4–22*^ + 7-NI primary cortical neurons. The image was captured at 60x magnification. Scale bar = 20μm. **C-D** Statistical analysis of mean fluorescence intensity of TSC2 within Camk2-α/PV positive neurons (n = 6). **E** Volcano plot analysis of the global cortical proteome comparing WT and Shank3^Δ4*–*22^ KO mice. The x-axis represents the log₂ fold change (FC) in protein expression between Shank3^Δ4*–*22^ KO and WT cortex, while the y-axis represents the −log₁₀(p-value). The horizontal dashed line indicates the statistical significance threshold (p = 0.05). Proteins plotted above this line are considered significantly differentially expressed, with positive and negative log₂FC values indicating upregulation or downregulation, respectively, in the Shank3^Δ4*–*22^ KO cortex (n = 3). **F** Representative western blot of total Herc1 in WT1, *Shank3*^Δ*4–22*^ KO and *Shank3*^Δ*4–22*^ KO + 7-NI. **G** Statistical analysis of the relative abundance of Herc1 (n = 5), in WT1, *Shank3*^Δ*4–22*^ KO and *Shank3*^Δ*4–22*^ KO + 7-NI. β-actin was used as a loading control **H** Representative western blot of ubiquitin, Herc1 and TSC2 after IP of TSC2 in WT1, *Shank3*^Δ*4–22*^ KO and *Shank3*^Δ*4–22*^ KO + 7-NI, IgG isotype control **I** Representative western blot of TSC2 and ubiquitin in WT1, *Shank3*^Δ*4–22*^ KO and *Shank3*^Δ*4–22*^ KO + MG132 primary cortical neuron (n = 4). **J-K** Statistical analysis of relative abundance of TSC2 and ubiquitin in WT, *Shank3*^Δ*4–22*^ KO and *Shank3*^Δ*4–22*^ KO + MG132 primary cortical neuron. Data are presented as mean ± SEM. A one-way ANOVA test with the Tukey’s multiple comparison’s test in all groups. *p < 0.05, **p < 0.01, ns not significant.

We observed a significant reduction in TSC2 expression in both excitatory and inhibitory neurons of *Shank3*^Δ*4–22*^ ASD model. Immunofluorescence analysis of primary cortical neurons from *Shank3*^*Δ4–22*^ mice revealed markedly decreased TSC2 fluorescence intensity in CaMKIIα-positive excitatory neurons as well as in parvalbumin (PV)-positive inhibitory neurons compared to wild-type controls. Treatment with the nNOS inhibitor 7-NI restored TSC2 levels in both neuronal subtypes (Fig. 3A-D). These findings indicate that nitric oxide-mediated S-nitrosylation and subsequent degradation of TSC2 occurs broadly across major neuronal populations. As mTOR is a master regulator of protein synthesis [65], we assessed global protein translation using anti-puromycin immunodetection. We have found altered global protein translation in *SHANK3 KO* SH-SY5Y cells compared to Control SH-SY5Y cells (Supplementary figs. S5A and S5B). 7-NI treatment for 24 h significantly prevented aberrant protein translation in *SHANK3* KO cells as compared to vehicle treated *SHANK3* KO cells (Supplementary figs. 5A and B). We have found no significant change in protein translation between control SH-SY5Y treated with 7-NI as compared to control SH-SY5Y treated with vehicle (Supplementary figs. S5A and B). Global proteomics was performed to study the changes in the protein expression landscape in the brains of the mutant mice. The MS data confirmed the alteration of the global protein translation in the *Shank3*^Δ*4–22*^ KO mice cortex compared to the WT cortex (Fig. 3E). Next, we performed an IP of TSC2, measured Ubiquitinated-TSC2 levels, and found increased ubiquitination of TSC2 in *Shank3*^Δ*4–22*^ KO mice compared to the WT controls. nNOS inhibition significantly prevented the ubiquitination of TSC2 in *Shank3*^Δ*4–22*^ KO mice treated with 7-NI (Fig. 3H). We next examined the interaction between TSC2 and the E3 ubiquitin ligase Herc1 [66]. Immunoblot analysis revealed a significant increase in the total level of Herc1 in *Shank3*^*Δ4–22*^ knockout mice compared to wild-type controls (Fig. 3F, G). Furthermore, co-immunoprecipitation of TSC2 followed by Herc1 immunoblotting demonstrated elevated TSC2-Herc1 association in *Shank3*^*Δ4–22*^ mice, indicating enhanced ubiquitination activity. Treatment with the nNOS inhibitor 7-NI effectively normalized total Herc1 expression and reduced its co-immunoprecipitation with TSC2, suggesting that nNOS inhibition mitigates Herc1-mediated ubiquitination and subsequent degradation of TSC2 (Fig. 3H). Further, to confirm increased ubiquitination of TSC2 leads to its proteasomal degradation we treated *Shank3*^Δ*4–22*^ primary cultured neurons with MG132. The *Shank3*^Δ*4–22*^ KO cells treated with MG132 show rescue in the total level of TSC2 as compared to *Shank3*^Δ*4–22*^ KO cells treated with vehicle (Fig. 3I, J. We have found no significant change in the ubiquitin level in *Shank3*^Δ*4–22*^ KO cells treated with MG132 as compared to *Shank3*^Δ*4–22*^ KO cells treated with vehicle (Fig. 3I, K). These results suggest that nNOS inhibition prevents TSC2 S-nitrosylation and its ubiquitination and subsequent degradation.

### Overactivation of mTOR leads to an ASD-like phenotype

To examine the effects of mTOR activation on behavioral phenotypes related to ASD, we performed a set of behavioral tests following treatment with TG003 [67], an mTOR signaling pathway activator. We have conducted behavioral tests on C57BL/6 J (WT) mice and C57BL/6 J mice treated with TG003 (WT + TG003). All mice in both groups were 6–8 weeks old male mice.

**Fig. 4:**
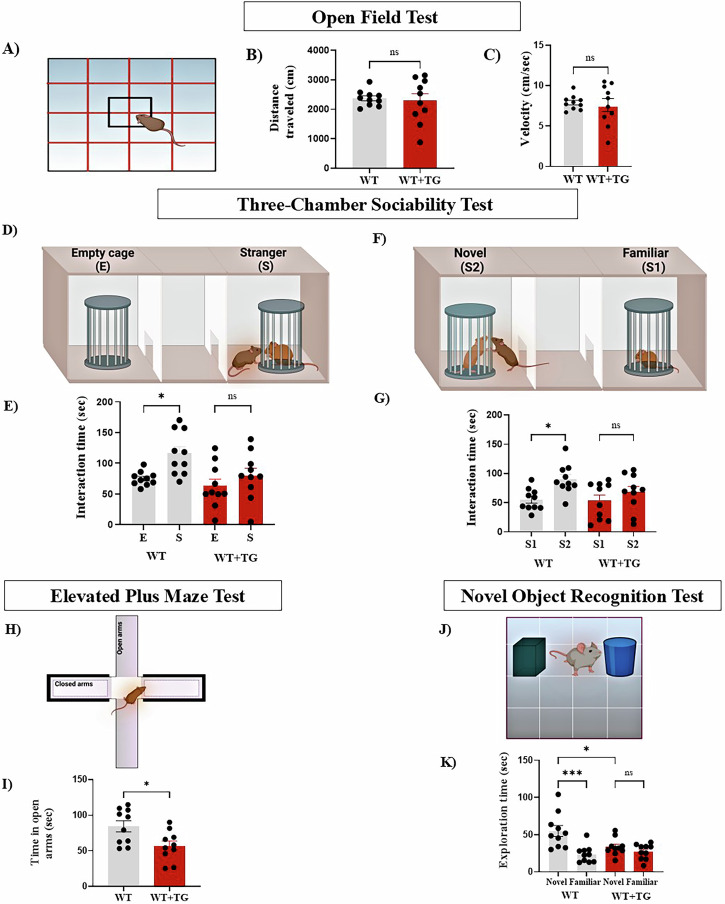
mTOR overactivation can lead to ASD-like behavior in WT mice. Behavioral tests analysis was conducted for the following groups of male mice: 1. WT and WT + TG003. **A** An illustration of an open field test platform. **B-C** Statistical analysis of the distance travelled (in cm) and velocity of mice (in cm/sec) in WT and WT + TG003. The data are presented as the mean ± SEM. Statistical significance was determined using an unpaired two-tailed Student’s t-test, ns = not significant. **D** An illustration of three-chamber sociability test platform (the first session) and (**E**) The statistical analysis of the interaction time with either an empty cage (**E**) or a stranger mouse. The data are presented as the mean ± SEM. One-way ANOVA followed with the Tukey’s multiple comparison test in all groups. *p < 0.05, ns not significant. **F** An illustration of three-chamber sociability test platform (the second session) and (**G**) The statistical analysis of the interaction time with either a familiar mouse (S1) or a novel mouse (S2). The data are presented as the mean ± SEM. One-way ANOVA followed with the Tukey’s multiple comparison test in all groups. *p < 0.05, ns not significant. **H** An illustration of the elevated plus maze test platform. **I** Statistical analysis of the time spent in the open arms. The data are presented as the mean ± SEM. Statistical significance was determined using an unpaired two-tailed Student’s t-test, *p < 0.05. **J** An illustration of Novel object recognition (NOR) test platform. **K** Statistical analysis of the object exploration time of either a novel (N) or a familiar (F) object. The data are presented as the mean ± SEM (n = 10). One-way ANOVA followed with the Tukey’s multiple comparison test in all groups. *p < 0.05, ***p < 0.001, ns not significant.

Initially, we performed the open field test to measure the distance travelled and velocity, and we found no significant change in both groups WT compared to WT treated with TG003 (Fig. 4A-C). In the three-chamber sociability test, the vehicle-treated WT mice spent significantly more time interacting with the stranger mouse as compared to the empty cage, reflecting an intact sociability, however, the WT treated mice (WT + TG003) failed to exhibit any significant interest in interacting with the stranger mice or empty cage, indicating sociability deficits (Fig. 4D, E). On the second day of the test, the WT mice treated with TG003 showed interest neither in stranger nor familiar mice (Fig. 4F, G). In the elevated plus maze experiment, we found that WT mice treated with TG003 spent less time in the open arm than WT mice treated with vehicle showing anxious behavior in WT + TG003 treated mice (Figs. 4H I). Finally, we conducted novel object recognition tests and found that WT mice treated with TG003 showed no particular interest in the novel or familiar object showing ASD-like phenotype. In contrast, WT mice explored more time with novel objects than familial objects (Fig. 4J, K). These behavioral assays suggest that overactivation of mTOR with TG003 leads to the ASD-like behavioral phenotype similar to those observed in *Shank3*^Δ*4–22*^ and *Cntnap2*^(-/-)^ mice.

### The C203S mutation in TSC2 prevents its S-nitrosylation and reverses mTOR activation and ASD-like phenotypes in both in vitro and in vivo models

**Fig. 5:**
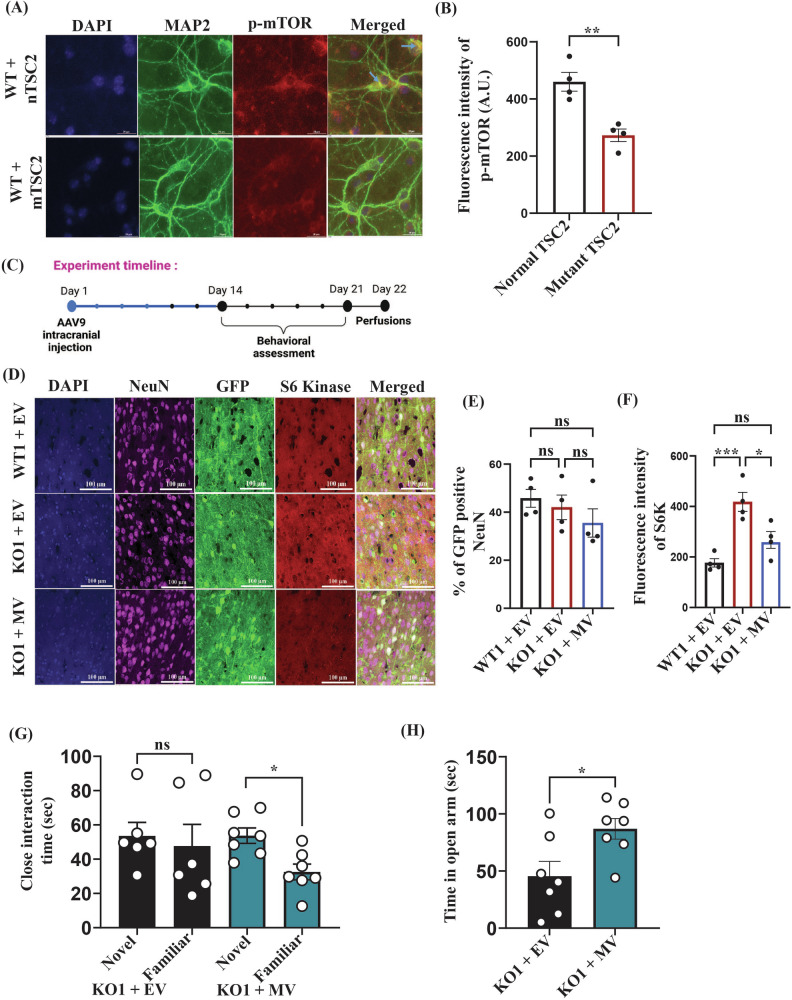
The C203S mutation in TSC2 prevents its S-nitrosylation and reverses mTOR activation and ASD-like phenotypes in both in vitro and in vivo models. **A** Representative confocal images of DAPI (blue), MAP2(Green), and p-mTOR(red) in WT cortical neurons overexpressed with WT TSC2 and mutated TSC2-C203S plasmid. The image was captured at 60x magnification. Scale = 20μm. **B** Statistical analysis of fluorescence intensity of p-mTOR. The data are presented as the mean ± SEM (n = 4). Statistical significance was determined using an unpaired two-tailed Student’s t-test, **p < 0.01. **C** Experimental timeline for the AAV injection in the mice and doing behavioral and biochemistry experiments. **D** Representative confocal images of DAPI (blue), GFP(Green), p70S6 kinase(red) and NeuN (purple) in *Shank3* WT overexpressed with EV (empty vector), *Shank3*^Δ*4–22*^ KO overexpressed EV (empty vector) and *Shank3*^Δ*4–22*^ KO overexpressed with MV (mutant vector, C203S). The image was captured at 60x magnification. Scale = 100μm. **E** Statistical analysis of neurons showing GFP positive. **F** Statistical analysis of fluorescence intensity of p70S6Kinase in GFP positive neurons (n = 4). **G** The statistical analysis of the interaction time with either a familiar mouse or a novel mouse of *Shank3*^Δ*4–22*^ KO overexpressed EV and *Shank3*^Δ*4–22*^ KO overexpressed with MV. Data are presented as mean ± SEM. A one-way ANOVA test with Tukey’s multiple comparison tests in all groups. *p < 0.05, ***p < 0.001, ns not significant. **H** Statistical analysis of the time spent in the open arms of *Shank3*^Δ*4–22*^ KO overexpressed EV and *Shank3*^Δ*4–22*^ KO overexpressed with MV. The data are presented as the mean ± SEM. Statistical significance was determined using an unpaired two-tailed Student’s t-test, *p < 0.05.

To test the biochemical effect of S-nitrosytable cysteine residues, we did site-directed mutagenesis of cysteine 203, a known prone S-nitrosytable site. We overexpress the mutated *TSC2* (m*TSC2*) and non-mutated *TSC2* (*nTSC2*) in WT cortical neurons. Neurons expressing mTSC2 show significantly lower p-mTOR fluorescence intensity than those expressing nTSC2 after exposure to SNAP (Fig. 5A, B). Next, to test the significance of the C203S residue at the biochemical and behavioral level, we injected the *mTSC2* packed in AAV9 stereotaxically in the prefrontal cortex of *Shank3*^Δ*4–22*^ KO mice (Supplementary figs. S7A and B).

We have found a significant decrease in the fluorescence intensity of p-mTOR of *Shank3*^Δ*4–22*^ injected with mTSC2 as compared to *Shank3*^Δ*4–22*^ injected with an empty vector (Supplementary figs. S8A and C). We found reduced p70S6 Kinase fluorescence intensity in *Shank3*^Δ*4–22*^ KO mice injected with mTSC2 compared to those injected with an empty vector (Fig. 5D-F). Next, we conducted behavioral tests. In three-chamber sociability test, in the first session of the sociability test, we did not find any significant interest in interacting mice/empty cage in both groups of *Shank3*^Δ*4–22*^ KO injected with mTSC2 or empty vector (Supplementary fig. S9). On the second day, we found that *Shank3*^Δ*4–22*^ KO mice injected with AA9-*mTSC* interacted more with the stranger mice than the familiar mice whereas *Shank3*^Δ*4–22*^ KO mice injected with AA9-GFP showed no significant interest in familiar/stranger mice (Fig. 5G). We have also found that the *Shank3*^Δ*4–22*^ KO mice injected with *mTSC2* were spending more time in the open arm than *Shank3*^Δ*4–22*^ KO mice injected with AAV9-GFP (Fig. 5H). The behavioral tests demonstrated that S-nitrosylation of C203 in TSC2 is critical for regulating social memory and anxiety-like behavior. We next examined whether restoring TSC2 expression via AAV9-mTSC2 could rescue synaptic deficits in Shank3^Δ4*–*22^ KO mice. Immunoblot analysis showed a marked increase in synaptophysin levels in AAV9-mTSC2-injected Shank3^Δ4*–*22^ KO mice compared to AAV9-GFP controls (Supplementary figs. S10A and B), indicating that mTSC2 expression restores synaptic integrity. The findings highlight the crucial role of C203 in TSC2 S-nitrosylation and function.

### Reduced TSC2 and elevated mTOR signaling in children with *SHANK3* mutations and idiopathic ASD

**Fig. 6:**
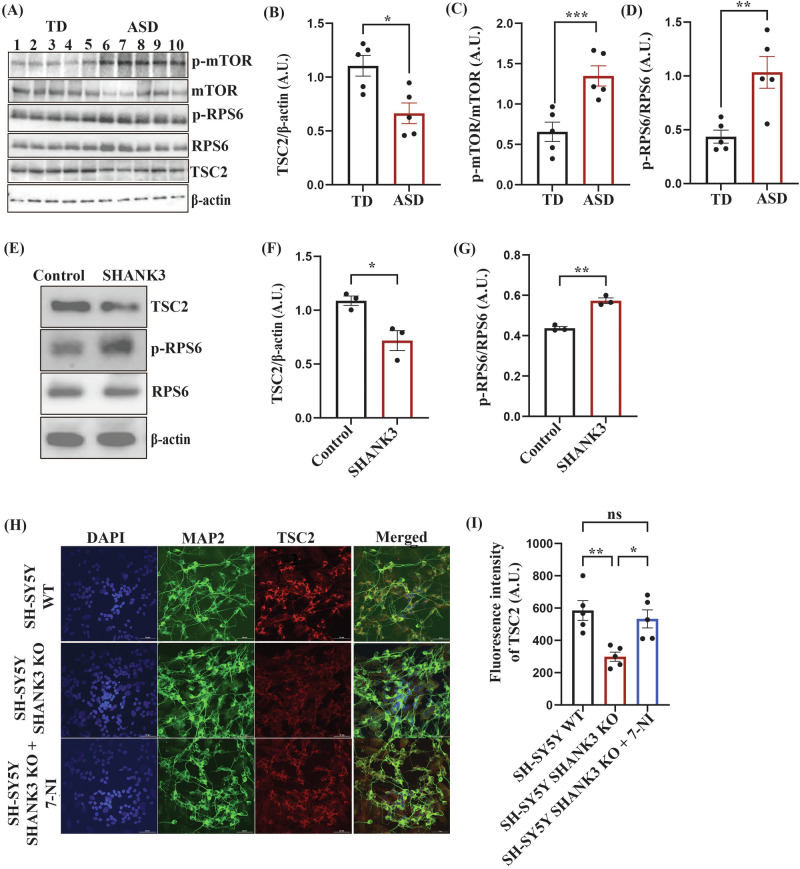
Relationship to human ASD samples. **A** Representative WB images of p-mTOR, mTOR, p-RPS6, RPS6 and TSC2 in TD (typically developed (1–5)) and children with ASD (6–10). β-actin was used as a loading control. **B-D** Statistical analysis of relative abundance of TSC2/β-actin (n = 5), p-mTOR/mTOR (n = 5), p-RPS6/RPS6 (n = 5) in TD (typically developed) and children with ASD. The data are presented as the mean ± SEM. Statistical significance was determined using an unpaired two-tailed Student’s t-test, *p < 0.05, **p < 0.01, ***p < 0.001. **E** Representative WB images of p-RPS6, RPS6 and TSC2 in children with ASD due to SHANK3 LOF and their parents as the control group. β-actin was used as a loading control. **F-G** Statistical analysis of relative abundance of p-RPS6/RPS6 (95% Confidence interval = 0.09085–0.1804) and TSC2 (95% Confidence interval = −0.6536–−0.08673) (n = 3) in children with ASD due to SHANK3 LOF and their parents as the control group. The data are presented as the mean ± SEM. Statistical significance was determined using an unpaired two-tailed Student’s t-test, *p < 0.05, **p < 0.01. **H** Representative confocal images of DAPI (blue), MAP2(Green), and TSC2(red) in Control SH-SY5Y (WT), *SHANK3* KO SH-SY5Y cells, and *SHANK3* KO SH-SY5Y cells + 7-NI. The image was captured at 60x magnification. The scale bar is 50μm. **I** Statistical analysis of fluorescence intensity of TSC2. A one-way ANOVA test with Tukey’s multiple comparison tests in all groups. *p < 0.05, **p < 0.01 and ns not significant.

To investigate the relevance of our findings in humans, we analyzed mTOR signaling proteins in plasma samples from children with ASD and age-matched typically developing (TD) controls. We have found that there is an increase in p-mTOR and p-RPS6 in the ASD plasma samples as compared to the TD group (Fig. 6A, C and D). We have also found decreased levels of TSC2 in the ASD samples compared to TD ones (Fig. 6A, B). We further tested the above proteins in the plasma samples of kids with *SHANK3* mutations as compared to their control parents. We have found a decreased level of TSC2 in kids with ASD due to *SHANK3* LOF as compared to the control parents (Fig. 6E, F). We have also found significantly increased level of p-RPS6 in kids with *SHANK3* LOF as compared to their parents (Fig. 6E, G). Further, we also measured fluorescence intensity in the differentiated human control SH-SY5Y and *SHANK3* KO SH-SY5Y cells. We found a decreased fluorescence intensity of TSC2 in *SHANK3* KO SH-SY5Y cells. 7-NI treatment for 24 h rescued the level of TSC2 in *SHANK3* KO SH-SY5Y cells (Fig. 6H, I).

## Discussion

**Fig. 7:**
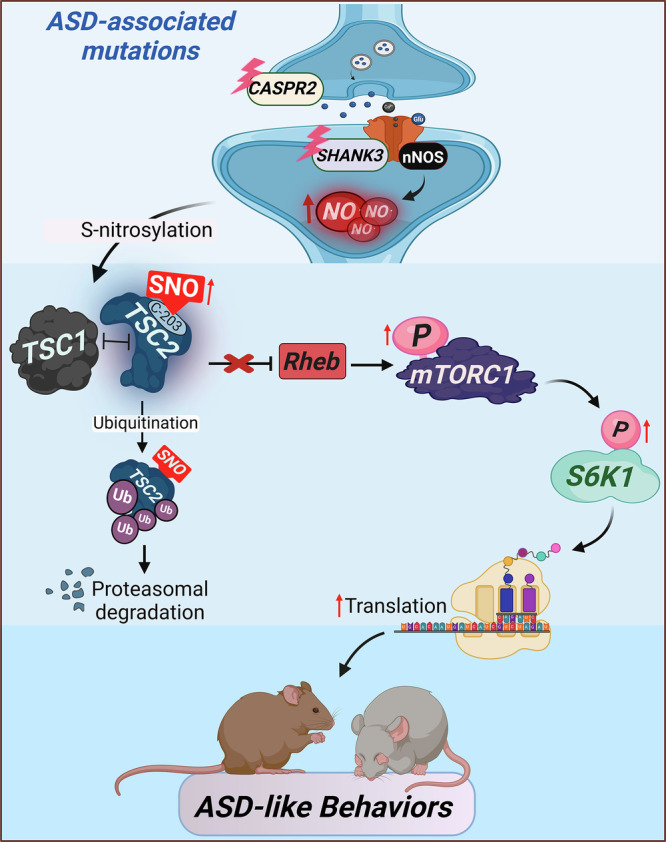
Mechanistic illustration of the SNO-TSC2-dependent mTOR activation. Increased S-nitrosylation of TSC2 promotes its proteasomal degradation, leading to aberrant mTOR overactivation. Inhibition of TSC2 S-nitrosylation restores TSC2 stability, normalizes mTOR signaling, and rescues ASD-like phenotypes.

Our study provides strong evidence that dysregulation of the mTOR signaling pathway specifically mediated through S-nitrosylation of TSC2, plays a critical role in the pathogenesis of ASD. We showed that elevated NO levels induce increased S-nitrosylation of TSC2, resulting in its destabilization and subsequent degradation, thereby driving hyperactivation of the mTOR pathway. These findings were consistently observed in two established ASD mouse models, *Shank3*^Δ*4–22*^ and *Cntnap2*^(-/-)^. To confirm NO dependence and exclude off-target effects, we pharmacologically inhibited distinct NOS isoforms in Shank3^Δ4*–*22^ KO mice (Supplementary Fig. S6). nNOS blockade with 7-NI and non-selective NOS inhibition with L-NAME significantly restored cortical TSC2 toward WT levels, whereas the iNOS-selective inhibitor L-NIL produced no significant change relative to KO + vehicle. These data implicate nNOS-derived NO in the TSC2 reduction observed in the KO mice (Fig. 7).

The mTOR signaling pathway is a central regulator of diverse cellular processes, including protein synthesis, autophagy, synaptogenesis, and neuronal metabolism [68–71]. Dysregulation of this pathway has been associated with a range of neurological and developmental disorders, particularly ASD [13, 43, 48, 68]. Previous studies have consistently identified aberrant mTOR signaling in ASD, characterized by increased phosphorylation of downstream targets such as ribosomal protein S6 (RPS6) and eukaryotic translation initiation factor 4E-binding protein 1 (4E-BP1) [46, 72–74]. Indeed, our findings further validate the elevated levels of phosphorylated mTOR (p-mTOR) and phosphorylated RPS6 (p-RPS6) observed in *Shank3*^Δ*4–22*^ and *Cntnap2*^(-/-)^ mice, reinforcing the critical role of mTOR hyperactivity in ASD pathophysiology. Significantly, while earlier reports documented the overactivation of the AKT-mTOR pathway in *Cntnap2*^(-/-)^ mouse models, the underlying molecular mechanisms remained poorly defined [75]. Previous studies suggest that oxidative and nitrosative stress can also modulate multiple upstream cascades (PI3K-Akt, AMPK, and ERK/MAPK) to mTOR1. For example, p38 MAPK and ERK pathways are known to interact with mTOR through TSC2 and REDD1 regulation, while AMPK responds to metabolic stress to inhibit mTOR activity[76]. Future work integrating these pathways will be valuable to comprehensively understand how NO-driven redox signaling converges on mTOR and contributes to ASD-related pathophysiology.

Emerging evidence indicates that S-nitrosylation, mediated by NO, plays an important regulatory role in mTOR signaling [77]. In our previous SNO-proteomics (SNOTRAP-based) study, we identified additional S-nitrosylated proteins involved in the mTOR pathway, including TSC2, mLST8, p38MAPK, suggesting a broader redox-sensitive regulation of this signaling network [36]. In the context of TSC2, such dysregulation leads to aberrant neuronal growth, synaptic changes, and defective autophagy. Our study provides novel insight by pinpointing S-nitrosylation of TSC2 as a key mechanism underlying this dysregulation. TSC2 functions as an essential negative regulator of mTORC1 by forming a complex with TSC1 to inhibit mTORC1 activity [68, 74]. Importantly, mTOR pathway overactivation is not unique to TSC but represents a shared hallmark among several neurodevelopmental disorders collectively termed mTORopathies including FXS, PTEN-associated autism, and NF1-related syndromes etc [43–45]. In these conditions, genetic or signaling perturbations converge on enhanced mTORC1 signaling, impairing synaptic plasticity and protein homeostasis. Therefore, SNO-induced inhibition of TSC2 offers a plausible redox-dependent mechanism that could exacerbate or mimic such hyperactivation even in the absence of canonical genetic mutations as shown in Fig. 2, with the treatment of SNAP (NO donor in WT mice. Indeed, aberrant S-nitrosylation has been implicated in broad neurodevelopmental and neurodegenerative contexts, suggesting that nitrosative stress may phenocopy genetic mTOR dysregulation [47, 78]. We therefore propose that SNO-mediated TSC2 modulation constitutes a potentially unifying mechanism across mTORopathies. This raises the possibility that redox modulation or targeted de-nitrosylating strategies might serve as therapeutic avenues to restore proper TSC2 function and normalize mTOR signaling. Future studies should explore whether SNO-TSC2 interactions are detectable in patient-derived models of FXS, PTEN-ASD, and NF1, thereby extending the translational relevance of these findings beyond TSC.

Our results provide compelling evidence that NO-mediated dysregulation of the mTOR pathway in ASD involves aberrant protein translation. Aberrant protein translation is increasingly recognized as a hallmark of ASD. Studies show that several ASD-related mutations converge on signaling pathways that regulate translation, notably the mTOR pathway, which controls the synthesis of new proteins at synapses [79] [80]. Dysregulation of this pathway can lead to excessive or defective protein synthesis, potentially disturbing synaptic development and plasticity, which are critical for normal social and cognitive behaviors [80]. In Shank3 KO mice, which model ASD-like behaviors [81], increased mTOR signaling has been observed [54]. This is consistent with findings that Shank3 normally interacts with key translation regulatory proteins. Loss of ASD gene disrupts these interactions, leading to hyperactivation of the mTOR pathway and increased protein translation [80]. They demonstrated that eIF4E-transgenic mice have increased protein translation, as measured by anti-puromycin immunoblotting, and that this is associated with hyperactive mTOR signaling [80]. We also assessed global protein translation using anti-puromycin labeling [82]. It has been reported that in pathological states, excessive or aberrant S-nitrosylation can impair protein function and contribute to neurodegeneration [83, 84]. For instance, in Alzheimer’s disease, S-nitrosylation of proteins such as dynamin-related protein 1 (Drp1) leads to mitochondrial fragmentation and neuronal injury [85]. Similarly, in Parkinson’s disease, S-nitrosylation of parkin impairs its E3 ubiquitin ligase activity, disrupting protein homeostasis and neuronal survival [86]. To investigate the mechanistic link between NO signaling and TSC2 stability, we performed immunoprecipitation for TSC2 and measured ubiquitin and Herc1 levels. Our result also showed that increased S-nitrosylation of TSC2 leads to increased ubiquitination of TSC2 through Herc1 E3 ligase activity and its proteasomal degradation. This parallel suggests the broader physiological significance of NO-dependent post-translational modifications in disease processes.

Dysregulated mTOR signaling disrupts synaptic protein synthesis, contributing to impaired synaptic function and neural circuit abnormalities central to ASD phenotypes [37, 54, 61]. Evidence from prior studies supports the critical role of balanced mTOR signaling in neuronal homeostasis, emphasizing that hyperactivation and hypoactivation can lead to profound disruptions in neural connectivity and function [51]. Moreover, ASD-like behavioral phenotypes, including repetitive behaviors and social impairments, have previously been ameliorated by pharmacological inhibition of mTOR [40, 47, 75, 87]. Correspondingly, our findings provide strong behavioral evidence linking TSC2 S-nitrosylation with ASD pathogenesis. Specifically, overexpression of a mutant TSC2 variant (C203S), resistant to S-nitrosylation, effectively normalized mTOR hyperactivation and ameliorated social novelty deficits and anxiety-like behaviors in *Shank3*^Δ*4–22*^ mice. This proves the therapeutic potential of targeting S-nitrosylation sites within key regulatory proteins as a viable strategy to alleviate ASD symptoms.

Furthermore, we extended our mouse model findings to human clinical samples, observing similar alterations in plasma from ASD individuals including those with *SHANK3* mutations compared to typically developed controls or their parents. ASD plasma exhibited elevated p-mTOR levels and decreased total TSC2, supporting the potential of these molecular markers as clinically relevant biomarkers for ASD diagnosis and monitoring. Due to the limited sample size, we did not perform subgroup statistical analyses to correlate specific clinical features with molecular markers of nitrosative stress and mTOR signaling pathway. However, this will be considered in future work with larger, stratified cohorts to better understand whether the SNO-TSC2-mTOR axis is preferentially altered ASD subgroups.

Collectively, our study significantly advances the understanding of ASD pathogenesis by delineating a clear molecular link between elevated NO levels, TSC2 S-nitrosylation, and subsequent mTOR hyperactivation. Still, we believe that NO may modulate not only mTOR signaling but also additional molecular pathways. The NO-SNO-TSC2 axis represents a critical point within the signaling networks disrupted in ASD. Therapeutic interventions targeting NO signaling, specifically through inhibition of nNOS, hold considerable promise for mitigating mTOR pathway dysregulation and alleviating associated neurological and behavioral deficits in ASD. Future studies should explore these therapeutic strategies and assess their efficacy and safety profiles, ultimately translating mechanistic insights into substantial clinical improvements for ASD, including individuals with *SHANK3* mutations.

## Supplementary information

SUPPLEMENTAL MATERIAL

## Data Availability

Data is provided within the manuscript or supplementary information files.
