# Supplementary material for: Nitric Oxide-Mediated S-Nitrosylation of TSC2 Drives mTOR dysregulation across Shank3 and Cntnap2 Models of Autism Spectrum Disorder
Source: Mol Psychiatry. 2026 Feb 25;31(7):3710–25. doi: 10.1038/s41380-026-03514-6 (PMC13269142; doi:10.1038/s41380-026-03514-6)
Supplement: Supplementary file 1 — SUPPLEMENTAL MATERIAL [file 41380_2026_3514_MOESM1_ESM.pdf]

Supplementary Figure S1:

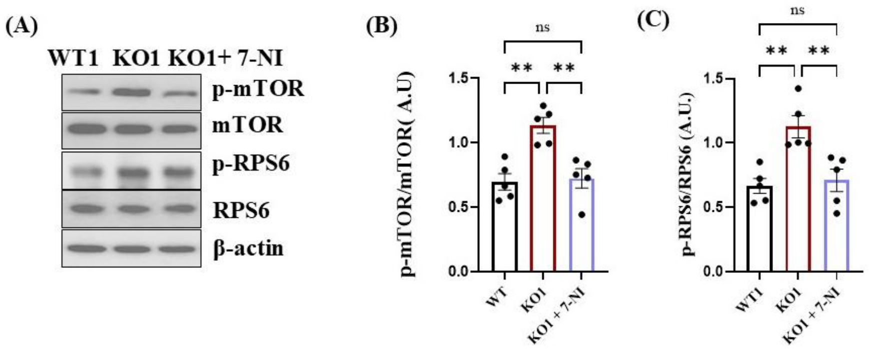

Supplementary Figure S1: 7-NI prevented overactivation of mTOR signaling pathways in striatum of *Shank3*$^{\Delta 4\text{-}22}$ KO. (A) Representative WB images of p-mTOR, mTOR, p-RPS6 and RPS6 in WT, *Shank3*$^{\Delta 4\text{-}22}$ KO and *Shank3*$^{\Delta 4\text{-}22}$ KO + 7-NI. β-actin was used as a loading control. (B-C) Statistical analysis of relative abundance of p-mTOR/mTOR (n=5), p-RPS6/RPS6 (n=5) in WT1, *Shank3*$^{\Delta 4\text{-}22}$ KO and *Shank3*$^{\Delta 4\text{-}22}$ + 7-NI. Data are presented as mean ± SEM. A one-way ANOVA test with the Tukey's multiple comparison's test in all groups. **p<0.01, ns = not significant.

Supplementary Figure S2:

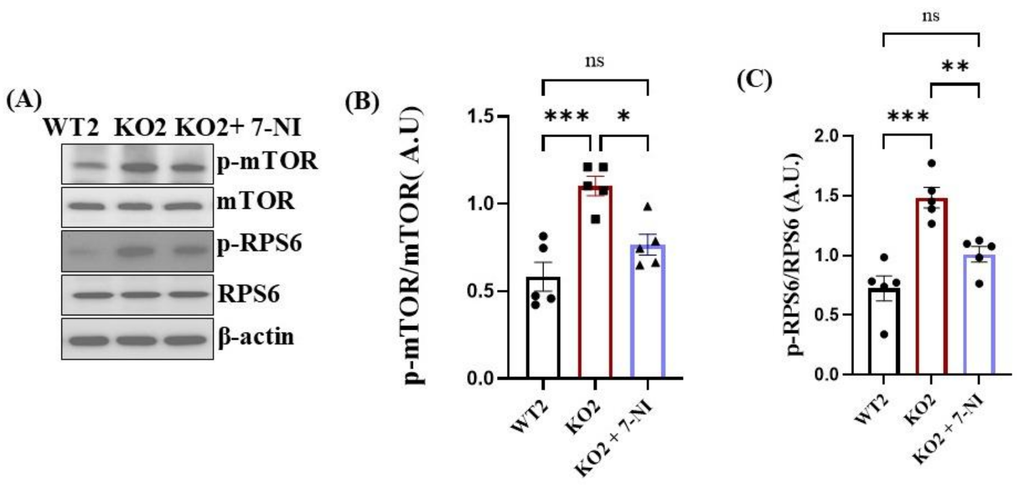

Supplementary Figure S2: 7-NI prevented overactivation of mTOR signaling pathways in *Cntnap2*$^{(-/-)}$ KO striatum. (A) Representative WB images of p-mTOR, mTOR, p-RPS6 and RPS6 in WT2, *Cntnap2*$^{(-/-)}$ KO and *Cntnap2*$^{(-/-)}$ KO + 7-NI. β-actin was used as a loading control. (B-C) Statistical analysis of relative abundance of p-mTOR/mTOR (n=5), p-RPS6/RPS6 (n=5) in WT2, *Cntnap2*$^{(-/-)}$ KO and *Cntnap2*$^{(-/-)}$ KO + 7-NI. Data are presented as mean ± SEM. A one-way ANOVA test with the Tukey's multiple comparison's test in all groups. *$p<0.05$, **$p<0.01$, ***$p<0.001$, ns = not significant.

Supplementary Figure S3:

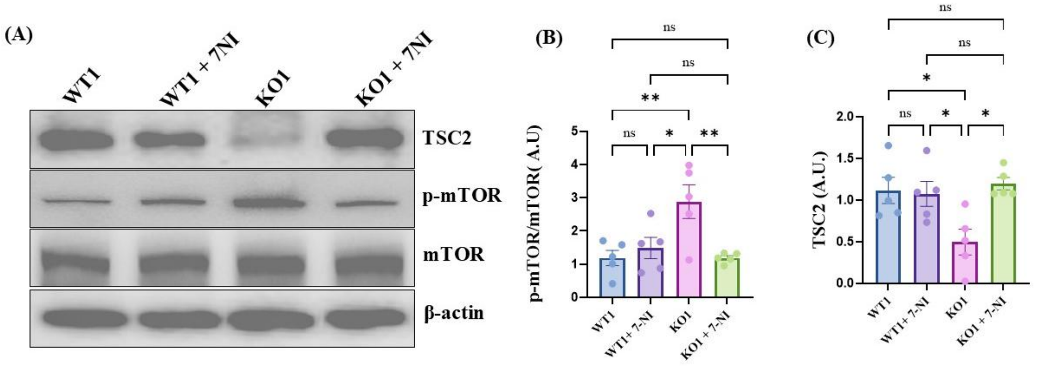

Supplementary Figure S3. Effect of 7-NI treatment on TSC2 and p-mTOR levels in WT and Shank3$^{\Delta 4-22}$ KO mice. (A) Representative immunoblots of TSC2, p-mTOR, mTOR in WT1+ vehicle, WT1 + 7-NI, *Shank3*$^{\Delta 4-22}$ KO + Vehicle and *Shank3*$^{\Delta 4-22}$ KO + 7-NI in cortical lysates. β-actin was used as a loading control. (B-C) Statistical analysis of relative abundance of p-mTOR/mTOR (n=5), TSC2 (n=5) in WT1+ vehicle, WT1 + 7-NI, *Shank3*$^{\Delta 4-22}$ KO + Vehicle and *Shank3*$^{\Delta 4-22}$ KO + 7-NI. Data are presented as mean ± SEM. A one-way ANOVA test with the Tukey's multiple comparison's test in all groups. *$p < 0.05$, **p<0.01, ns = not significant.

Supplementary Figure S4:

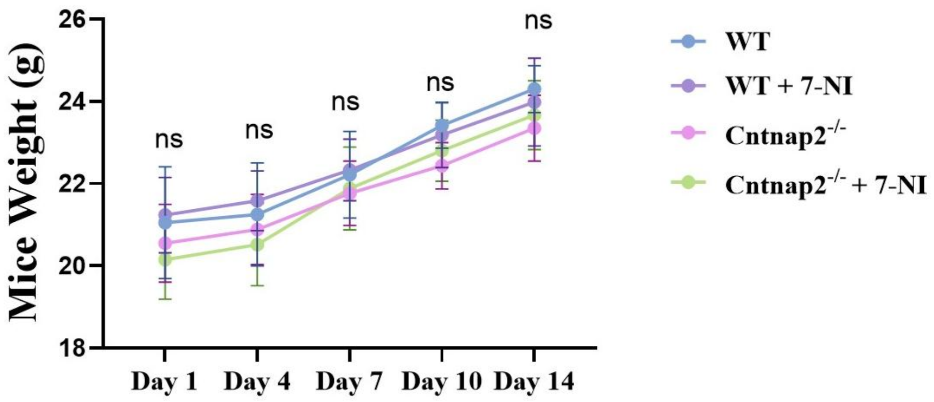

Supplementary Figure S4: 7-NI has no adverse effect on the body weight of mice. The body weight was measured on day 1, day 4, day 7, day 10 and day 14. There was no significant difference between the different groups WT + Vehicle, WT +7-NI, Cntnap2-

/- + vehicle and Cntnap2-/- +7-NI on the same days. A two-way ANOVA test with Tukey's multiple comparison tests in all groups. ns = not significant.

Supplementary Figure S5:

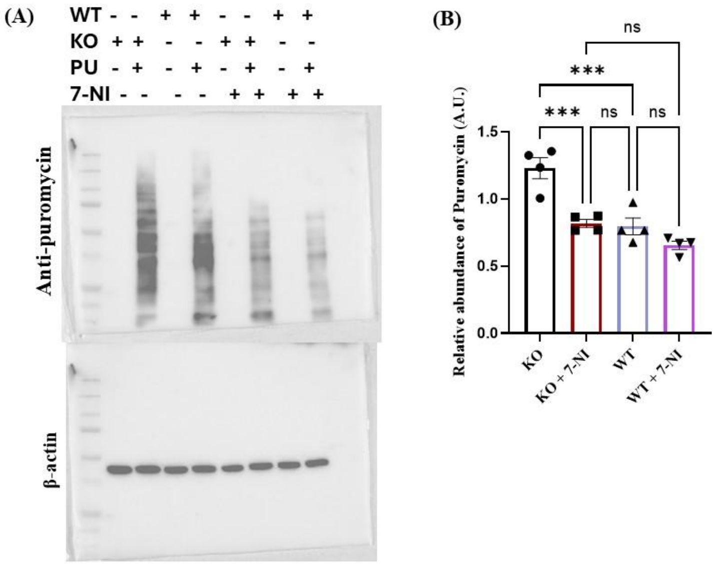

Supplementary Figure: S5. 7-NI prevented altered protein translation. (A) Representative WB images of Control SH-SY5Y(WT), *SHANK3* KO SH-SY5Y cells (KO), and *SHANK3* KO SH-SY5Y cells (KO) + 7-NI treated with puromycin. PU: Puromycin**, (**B) Statistical analysis of relative abundance of Control SH-SY5Y (WT), *SHANK3* KO SH-SY5Y cells (KO) and *SHANK3* KO SH-SY5Y cells (KO) + 7-NI treated with puromycin. β-actin was used as a loading control (N=4). A one-way ANOVA test with the Tukey's multiple comparison's test in all groups. ***$p<0.001$, ns= not significant.

Supplementary Figure S6:

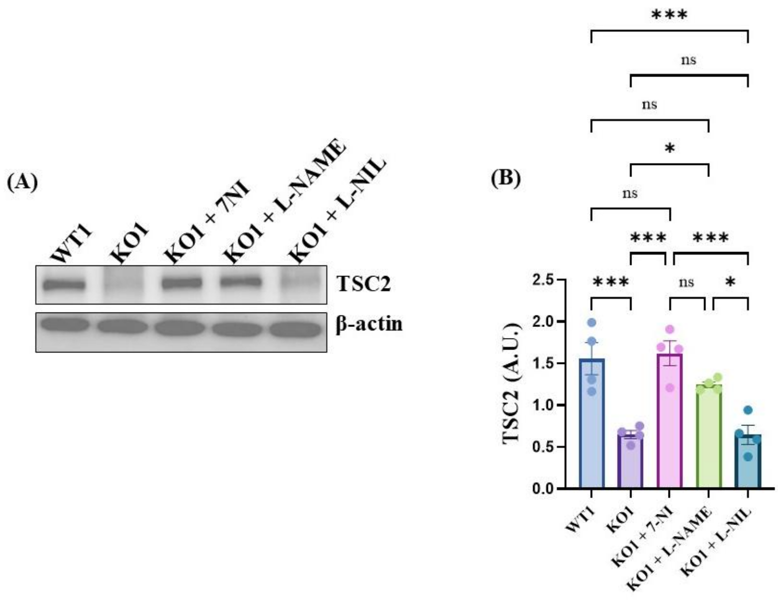

Supplementary Figure S6. To test the effect of different NOS inhibitors. (A) Representative immunoblots showing TSC2 cortical lysates from WT1, Shank3$^{\Delta4-22}$ KO, Shank3$^{\Delta4-22}$ KO + 7-NI, Shank3$^{\Delta4-22}$ KO + NAME, and Shank3$^{\Delta4-22}$ KO + L-NIL groups. (B) Statistical analysis of relative abundance of TSC2 (n=4) in WT1, Shank3$^{\Delta4-22}$ KO, Shank3$^{\Delta4-22}$ KO + 7-NI, Shank3$^{\Delta4-22}$ KO + NAME, and Shank3$^{\Delta4-22}$ KO + L-NIL. β-actin was used as a loading control. Data are expressed as mean ± SEM, A one-way ANOVA test with the Tukey's multiple comparison's test in all groups. *$p < 0.05$, ***p<0.001, ns = not significant.

Supplementary Figure S7:

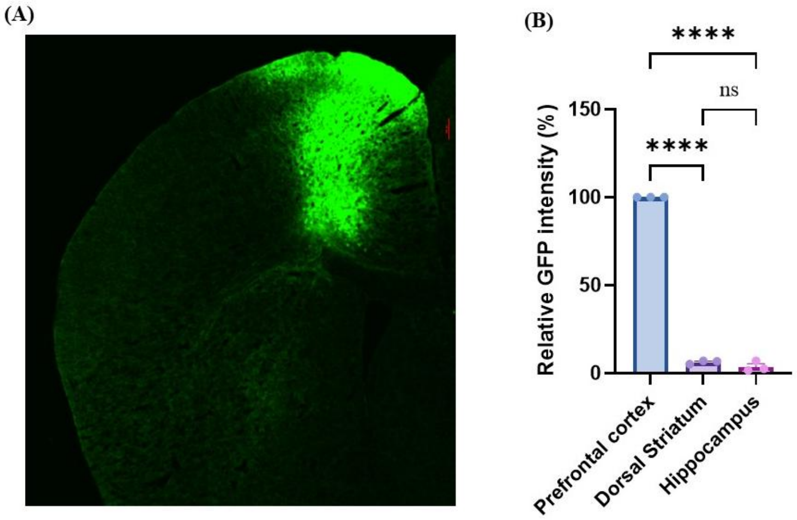

Supplementary Figure S7. Post-hoc validation of AAV9-GFP expression in the mouse cortex. Representative confocal fluorescence image at 4x, showing AAV9-GFP expression in the targeted cortical region following stereotaxic injection. GFP fluorescence (green) indicates robust transduction at the one injection site with minimal diffusion to adjacent regions. (B) Quantitative analysis of GFP fluorescence intensity across brain regions. Scale bar = 200 μm. Data are presented as mean ± SEM; n = 3 mice. Statistical significance was determined by one-way ANOVA with Tukey's post hoc test. ****p<0.0001, ns=not significant.

Supplementary Figure S8:

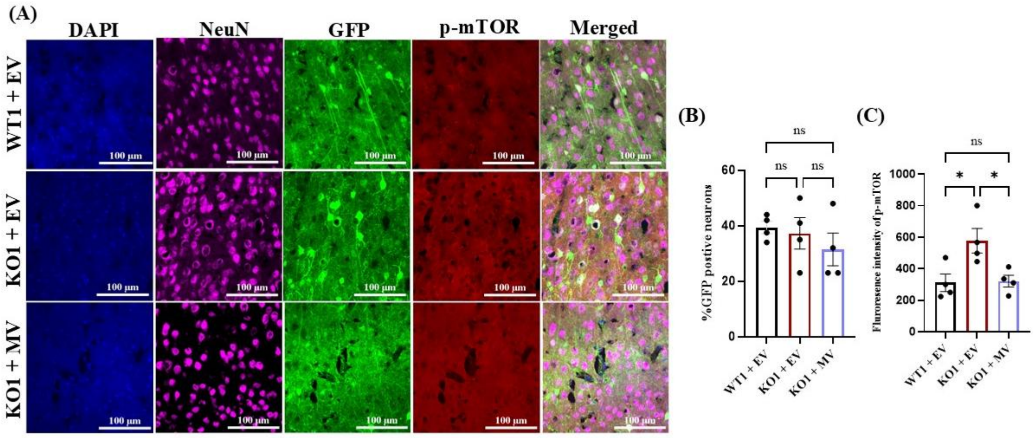

Supplementary Figure S8. The C203S mutation in TSC2 prevents its S-nitrosylation and mTOR overactivation in *Shank3*$^{\Delta 4\text{-}22}$ KO. (A) Representative confocal images of *Shank3* WT overexpressed with EV (empty vector), *Shank3*$^{\Delta 4\text{-}22}$ KO overexpressed with EV (empty vector) and *Shank3*$^{\Delta 4\text{-}22}$ KO overexpressed with MV (mutant vector, TSC2-C203S) showing DAPI (blue), GFP(Green), p-mTOR(red) and NeuN(purple). The image was captured at 60x magnification. Scale bar = 100μm. (B) Statistical analysis of neurons showing GFP positive. (C) Statistical analysis of fluorescence intensity of p-mTOR in GFP positive neurons. Data are presented as mean ± SEM, (n=4). A one-way ANOVA test with Tukey's multiple comparison tests in all groups. *p<0.05, ns = not significant.

Supplementary Figure S9:

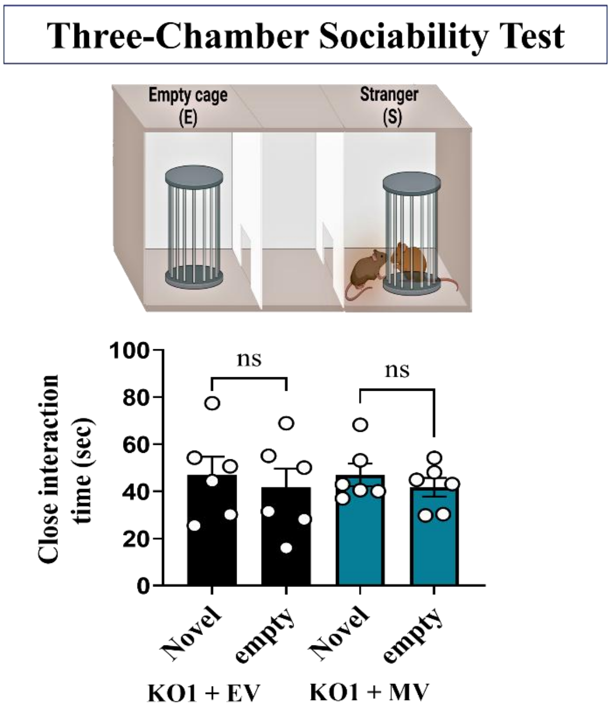

Supplementary Figure S9: C203S mutation playing a critical site in TSC2 S-nitrosylation and function. Behavioral test analysis was performed. A) Three Chambered sociability test in groups Shank3 KO + EV and Shank3 KO + MV. A one-way ANOVA test with Tukey's multiple comparison tests in all groups. ns = not significant.

Supplementary Figure S10:

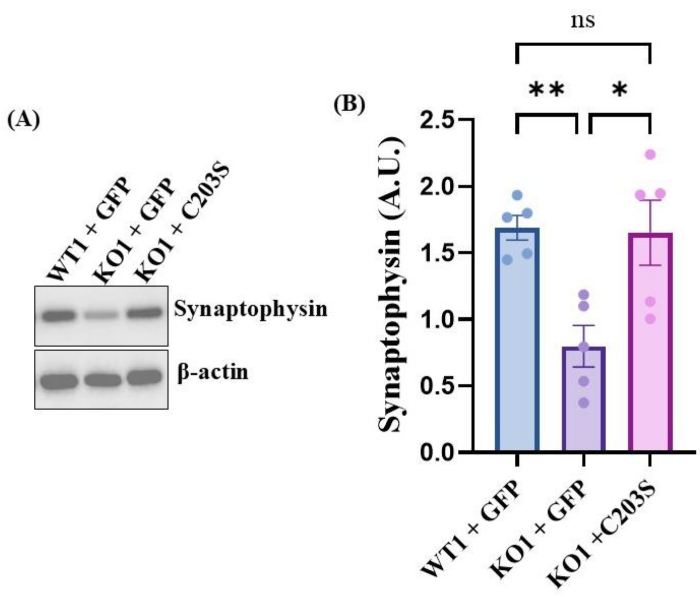

Supplementary Figure S10. Expression of mutant TSC2 restores synaptophysin levels in Shank3$^{\Delta 4\text{-}22}$ KO mice. (A)Representative WB images and (B) quantification showing synaptophysin level in cortical lysates from WT + GFP, Shank3$^{\Delta 4\text{-}22}$ KO + GFP, and Shank3$^{\Delta 4\text{-}22}$ KO + mTSC2 groups (n=5). β-actin was used as a loading control. Data are shown as mean ± SEM, A one-way ANOVA test with the Tukey's multiple comparison's test in all groups. *$p < 0.05$, **$p<0.01$, ns = not significant.

Supplementary Figure S11:

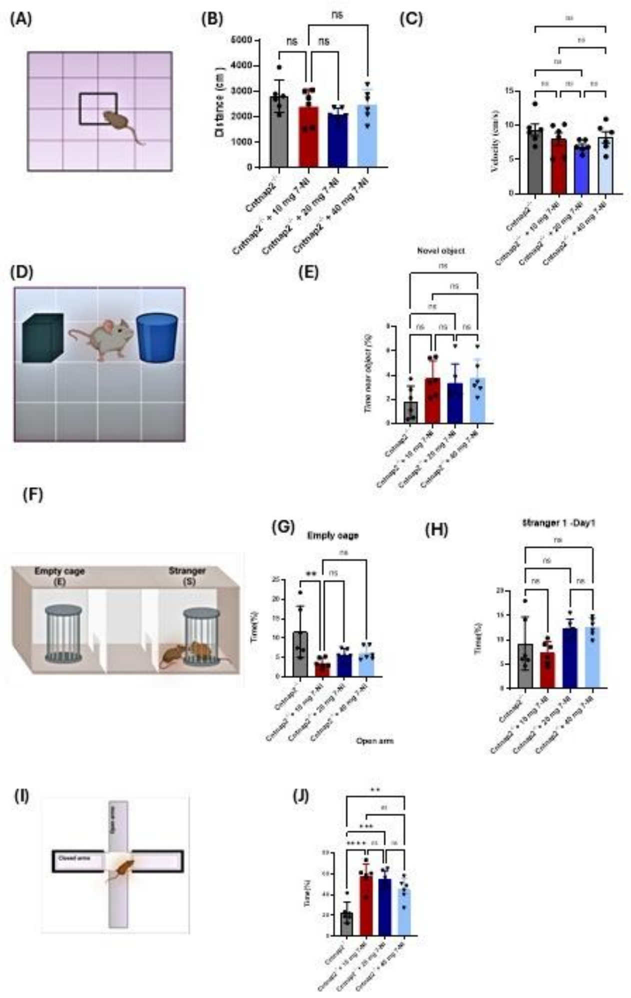

Supplementary Figure S11. Partial rescue of ASD-like phenotypes by low-dose nNOS inhibition in the Cntnap2$^{(-/-)}$ mice. Behavioral test analysis was conducted for the following groups of male mice: 1. Cntnap2$^{(-/-)}$ KO treated with vehicle, 2. Cntnap2$^{(-/-)}$ KO+ 10mg/kg, 3. Cntnap2$^{(-/-)}$ KO+ 20mg/kg, and 4. Cntnap2$^{(-/-)}$ KO+ 40mg/kg; i.p injection). A) Open Field test: an illustration of the test arena; (B) Statistical analysis of the distance traveled and (C) velocity in an open arena. D) Novel Object Recognition test: an illustration of the test arena; (E) Statistical analysis of the exploration time of a Novel object. F) Three-Chamber Sociability test: an illustration of the sociability phase (G-H) Statistical analysis of the interaction time with either an empty cage or a novel mouse. I) Elevated Plus Maze: an illustration of the maze test platform; (J) Statistical analysis of the time spent in the open arms. The data is presented as mean ± SEM. Statistical significance was determined using a One-way ANOVA followed with Tukey's multiple comparisons tests. **P < 0.01, ***P < 0.001, ****P < 0.0001, and ns = not significant.

Table S1: Clinical characteristics of the participants (TD and ASD).

TD/ASD	label (plasma)	age (months)	sex (Male =1)	cognitive impairment
TD1	C1P	59	1	-
TD2	C2P	38	1	-
TD3	C3P	64	1	-
TD4	C4P	62	1	-
TD5	25-P	68	1	-
ASD1	3P	58	1	mild-moderate
ASD2	4P	38	1	moderate
ASD3	1947	66	1	mild-moderate
ASD4	DN	66	1	mild-moderate
ASD5	95-p	25	1	Moderate

Table S2: Clinical characteristics of the participants (children with ASD due to SHANK3 LOF and their parent as Control).

Control/ASD	label (plasma)	age (years)	sex	cognitive impairment
Control 1	BH-Control	-	Father	-
Control 2	YZ-Control	-	Father	-
Control 3	MN-Control	-	Mother	-
SHANK3 ASD1	BH	6.6	Girl	severe
SHANK3 ASD2	YZ	9.4	Girl	moderate
SHANK3 ASD3	MN	6.1	Boy	severe
